# How nursing students learn infection control education through undergraduate nursing programs: a phenomenographic research study

**DOI:** 10.1186/s12912-023-01465-9

**Published:** 2023-08-31

**Authors:** Sung Ok Chang, Kyeong-Yae Sohng, Kyunghee Kim, Jongsoon Won, Seung-Kyo Chaung, Min-Jung Choi

**Affiliations:** 1 College of Nursing and BK21 FOUR R&E Center for Learning Health Systems, Korea University, Seoul, Republic of Korea; 2 College of Nursing, The Catholic University of Korea, 222 Banpo-daero, Seocho-Gu, Seoul, 06591 Republic of Korea; 3 Red Cross College of Nursing, Chung-Ang University, Seoul, Republic of Korea; 4 College of Nursing, Eulji University, Seongnam, Republic of Korea; 5 Department of Nursing, Semyung University, Jecheon, Republic of Korea

**Keywords:** Diploma programs, Education, Infection control, Nursing, Students, Phenomenography

## Abstract

**Background:**

Competency in infection control is crucial for implementing nursing best practices to ensure patient safety. However, research is lacking on the infection control education received by nursing students prior to entering clinical settings as nurses. This study aimed to explore how nursing students conceptualize infection control care in undergraduate nursing programs.

**Methods:**

This study employed a qualitative research method using phenomenography. Universities providing undergraduate nursing programs in Korea. Thirty nursing students: 10 students each from the 2^nd^, 3^rd^, and 4^th^ years of five undergraduate programs. Data were collected from May 2019 to February 2020 through semi-structured interviews and analyzed using a phenomenographic analysis procedure.

**Results:**

Six descriptive categories were derived inductively for nursing students’ frames of reference regarding infection control care and six descriptive categories of how nursing students learned about infection control care. The structural framework of the identified categories, about how nursing students learn about infection control care, was presented as an outcome space.

**Conclusions:**

Given that nursing students demonstrate diverse conceptualizations of infection control and are at varying levels of learning, professors and clinical mentors need to develop theoretical education and clinical practice opportunities that consider these differences.

**Supplementary Information:**

The online version contains supplementary material available at 10.1186/s12912-023-01465-9.

## Background

In 2022, the World Health Assembly approved the “Global Strategy on Infection Prevention and Control (IPC)” to address the silent burden of healthcare-associated infections and antimicrobial resistance [1]. IPC represents a practical, evidence-based approach to preventing avoidable infections and protecting patients and healthcare workers [2]. It encompasses various components such as infection prevention, including auditing, risk assessment, ongoing education, and infection control (IC) measures designed to manage the spread of infection and control outbreaks. Competency in IPC is crucial for implementing best nursing practices, ensuring patient safety, and delivering high-quality care [3, 4]. Despite numerous studies emphasizing the critical role of nurses in IC, assessing the IC education they receive before entering the workforce remains challenging [5–8].

Nursing students, who will play an essential role in the hospital, must be trained in IC [9, 10]. A previous study [8] found that nursing students demonstrated higher compliance with IC regulations based on factors such as their affiliated university, grade level, and experience with seminars/training on IC. Additional studies have reported inadequate IC education in nursing curricula [11–13]. Therefore, it is crucial to reorganize nursing curricula to better align with students' perspectives and integrate education that connects IC theory and practice to enhance nursing students’ understanding and compliance with IC measures [14, 15].

In the teaching process, students hold various conceptions that teachers try to change, modify, or successfully replace. The conceptions held by students differ from those that the teacher is trying to help the students construct; discrepancies have been reported to occur during the learning process [16]. Moreover, undergraduate nursing curricula generally incorporate IC education throughout students’ academic careers; however, dedicated IC courses are not offered [3, 6]. As suggested in a previous study, an integrated education approach can lead to changes in infection control education hours, which can affect nursing students' knowledge and attitudes toward IC. Therefore, integrated infection education can be done flexibly, which can affect the IC competency of nursing graduates. Considering the importance of infection control in clinical settings, dedicated education on IC is needed in the undergraduate nursing curriculum [6].

In addition, IC training in undergraduate nursing programs is not infused with a coherent approach that advances step-by-step throughout the curricula, which may compromise the development of safety-related nursing skills [10, 15]. Therefore, the degree to which the concept of infections is integrated throughout the undergraduate nursing curricula cannot be accurately ascertained [17].

Although the important role of nurses in IC is emphasized, only a few studies have investigated the IC education and training that nurses receive before entering the workplace [18, 19]. Moreover, IC learning has mainly been examined from the perspectives of nurses and researchers rather than students, and studies that conceptualize and explore learning from learners’ standpoints are quite rare. As they grow, people learn to conceptualize their own realities [16]; hence, it is necessary to explore how the nature of learning—as experienced by learners—varies according to context.

Therefore, this study aimed to explore how nursing students conceptualize and learn IC among IPC care in undergraduate nursing programs from the nursing students’ points of view.

## Methods

### Research design

As defined by Marton [20], phenomenography is the empirical study of the limited number of qualitatively different ways in which various phenomena and aspects of the world around us are experienced, conceptualized, understood, perceived, and apprehended. The origins of phenomenography lie in the approach to learning, the study of the different ways students think about and engage in learning [16]. The phenomenographic research approach can reveal qualitatively different ways people experience phenomena [21]. Applying this approach to nursing research explicitly allows for exploring nursing students' varying experiences, states, and needs. This study used phenomenography to focus on the learning phenomena experienced by learners. Phenomenography was utilized given the aim of the study because phenomenography is oriented toward discovering cognitive structures, that is, it entails the mapping of understanding into categories of meaning that individuals establish to arrive at specific conceptions about phenomena [16, 22]. This emphasis on differences in the concept of IC education implies that faculty can employ various measures or approaches to cater to the needs of different nursing students. Therefore, phenomenography was considered appropriate for this study as we aimed to describe the conceptualization and understanding of IC education among undergraduate nursing students.

### Participants

We recruited 30 undergraduate nursing students for purposive sampling. There are 202 undergraduate nursing programs in Korea as of 2021, of which 201 are 4-year undergraduate nursing programs. By 2022, all 202 undergraduate nursing programs are expected to offer 4-year bachelor’s degree programs [23]. The participants comprised 10 s-year, 10 third-year, and 10 fourth-year nursing students. The inclusion criteria were as follows: Second-year nursing students who had received IPC education during their fundamentals of nursing classes, third-year nursing students with clinical practice experience, and fourth-year nursing students who had completed more than three semesters of nursing college courses.

To accommodate the diversity of the students’ IC learning, we considered regional variations and different clinical practice hospitals. For this study, we recruited undergraduate nursing students from five universities (three in Seoul, one in Gyeonggi Province, and one in Chungcheong Province). Voluntary participants were recruited via a “recruitment for research” notice posted at each university, and 30 nursing students who expressed voluntary participation were selected based on the order of contact. There is no ideal sample size for phenomenographic research; however, previous studies have suggested that 15–20 individuals can reveal changes in experiences and perceptions without generating excessive data for analysis [24, 25]. In this study, we collected comprehensive data for analysis by including 30 participants. The researchers confirmed data saturation during the data collection process. Out of 30, 25 participants were women and five were men, with an average age of 22 years. We conducted semi-structured interviews using nine vignettes for each student. Therefore, 270 interviews were conducted with 30 students.

### Data collection

We collected data from May 2019 to February 2020 through semi-structured interviews, which were recorded using a voice recorder with participants’ consent. Interviews were conducted by researchers who were nursing educators at each of the five universities for a duration of 50–70 min per participant. The nursing educators explained to the students that they were free to answer the research questions and that this would not affect their grades. The nursing educator continued to listen while maintaining a comfortable atmosphere without evaluating or criticizing the students' responses so that the answers would not be hierarchical. The interviews were conducted individually in dedicated seminar rooms at the respective universities. These spaces were carefully selected to ensure an independent environment, free from noise interference, in accordance with the interview guidelines (see Additional File 1). We used patient care scenarios in nursing practice situations involving IC as a recall stimulus to collect participants’ conceptualizations of IC. To compose IC-related records, we requested that the infection control offices of three general hospitals located in Seoul, South Korea provide virtual cases related to infection in clinical practice. We collected 18 case scenarios; after discussions, we deleted similar parts and developed nine vignettes.

Table 1 shows the content of the nine vignettes for the three types of isolation. Carbapenem-resistant Enterobacteriaceae (CPE), Clostridium Difficile Infection (CDI), and Hepatitis A were selected as contact precaution cases, and Pertussis and Influenza A virus subtype (H1N1 Influenza) were selected as droplet precaution cases. Airborne diseases included tuberculosis (TB), Chickenpox, and Measles. We focused on understanding how nursing students conceptualize what they are taught regarding IC care and how they utilize their knowledge of IC care acquired throughout their undergraduate nursing program. We asked each participant to read the nine vignettes. We then asked the semi-structured interview questions (the interview guide is included as a supplementary file). These questions included the following:

- If you were a nurse, could you tell me what kind of nursing practice you would perform based on the scenarios related to the three types of isolation?
- What kind of nursing practices would you undertake to implement IC?
- What knowledge or experience is the foundation of current IC nursing practices?

**Table 1:** Nine vignettes on the types of infection transmission

Contact	Droplet	Airborne
Mr. Cho (M/70) Dx: Lung cancer O_2_: Ventilator Fever: 38.9°C Culture: CPE MICU care, severe diarrhea, antibiotics injection	Baby Song (M/10 m) Dx: Pertussis Ward care, conjunctivitis, mild fever, coughing with a distinctive "whooping" sound in inspiration and worse at night	Ms. Lee (F/45) Dx: Lung Tuberculosis Fever: 38.0°C Culture: TB Weight loss, anorexia, coughing
Ms. Cheon (F/51) Dx: Terminal ovarian cancer Fever: 38.0°C Culture: CDI ICU care, severe abdominal pain after chemotherapy	Ms. Yoon (F/29) Dx: Influenza (H1N1) Fever: 39.0°C ER admission, coughing, myalgia	Ms. Park (F/40) Dx: Acute myeloid leukemia, bone marrow transplantation (6 months ago) Fever: 38.7°C ER admission, itchy blister rash on trunk Newly diagnosed with Chicken pox
Mr. Kang (M/31) Dx: Hepatitis A (Anti-HAV IgM positive) Single-bed isolation room care, high fever, myalgia, diarrhea	Mr. Baek (M/11) Dx: Pertussis Isolation room care, coughing, rhinorrhea, sputum Nebulizer applied	Baby Kang (M/1 yr 6 m) r/o Measles Fever: 40.4°C Transferred to ward, general weakness, rash on neck, trunk, and extremities Measles IgM/IgG, PCR done, waiting for results

### Ethical considerations

The institutional review board of the university approved this research (No. SMU-2019–04-004). Prior to commencing the study, the research objectives, methodology, and the process of recording and transcribing the interviews were explained to the students. They were informed that the collected data would be strictly employed for research purposes and assured of their right to decline to respond to specific questions or withdraw their participation at any stage of the study. Written informed consent was obtained from all participants. The recordings and transcriptions were anonymized and securely saved on a password-protected device to safeguard confidentiality. The transcriptions were kept separately to ensure restricted access, with only the researcher having authorized access to the data. All participants were offered a gift certificate as a reward.

### Data analysis

Conceptions of reality are considered categories of description used to facilitate the understanding of concrete cases of human functioning [16]. We performed a phenomenographic data analysis to explore the participants’ categories of descriptions regarding IC and IC learning.

We used the phenomenographic analysis procedure described by L-O Dahlgren and M Fallsberg [26] and examined the data using the following seven steps: (1) Familiarization, (2) compilation, (3) condensation, (4) grouping, (5) articulation, (6) constructing a suitable linguistic expression, and (7) contrasting. To familiarize ourselves with the interview data, we thoroughly reviewed it multiple times to gain a comprehensive understanding. We engaged in a comparative analysis of the meaning units, aiming to identify qualitative variations encompassing differences and similarities in nursing students' experiences. Through condensation, we organized the significant statements into larger units, providing an explanatory framework for understanding the aspects of IC care and learning among nursing students. We then grouped these units based on similar experiences, revealing emerging patterns within each group of answers. This articulation process involved the utilization of suitable linguistic expressions in labeling the various categories that had been constructed. Finally, we compared the obtained categories, examining their similarities and differences. Through these steps, we identified the nursing students’ conceptual categories of IC care and IC learning in undergraduate nursing programs.

### Credibility

The core question of credibility in a phenomenographic study is about the relationship between the empirical data and the categories for describing the ways of experiencing a certain phenomenon [27].

To ensure the study’s credibility, we chose a method to explain the differences and similarities supported by empirical data. All the authors, who are nursing educators, continuously discussed their interpretations with each other at every stage of the analysis. Furthermore, all authors scrutinized any inconsistencies in the interview data to avoid overlooking ambiguities in understanding the nursing students’ conceptualizations of IC education in undergraduate nursing programs.

## Results

The result of a phenomenographic study is a set of categories describing the qualitative variations in the empirical material [28]. Therefore, we proposed the following categories of description: (1) What is your frame of reference for IC care in this situation? (2) How did you learn about the IC care for this situation? and (3) What is the structural framework of the identified categories concerning how nursing students learn IC care?

*CFs (categories of frame of reference)* were categorized as *CF1 to CF6* in the context of an organized image of caring for an infected patient in clinical practice as a nursing student's frame of reference for IC care. According to how nursing students learned about IC, *CLs (categories of learning)* were grouped into *CL1 to CL6*.

### What is your frame of reference for IC in this situation?

Nursing students' frame of reference for IC nursing is the category upon which nursing students are based performing infection control in the context of caring for infected patients. We identified six categories. Key quotes of participants from each category are presented in Table 2.

**Table 2:** Relevant quotations from interviews leading to the description categories on infection control by nursing students

Categories		Relevant quotations from interviews
Nursing students' frames of reference on IC care	CF1. Structuring the spatial separation of infected patients	The reason for quarantine in a single room is that there is a possibility of the pathogen spreading around. I thought it was important to separate the patient. I told you that pertussis is related to a drop precaution, as I know; however, I thought it best to keep a certain distance because it does not move through the air for a certain period of time, unlike infection through the air (a 2^nd^-year nursing student)
	CF2. Patterning IC protocol according to the pathogenic characteristics	First, the patient is informed that they are contagious because they came with symptoms and have been diagnosed with the new influenza. Therefore, after explaining that it is necessary to move to a single room, it was essential to wear a mask as the patient was contagious. After educating, I thought about allowing the patient to wear a mask and not making contact with other patients, especially older patients (a 2^nd^-year nursing student)
	CF3. Identifying the possibility of infection during the incubation period	I think it is necessary to investigate the people who have been in contact with the patient, and as 3 weeks is a long time, we should check the incubation period of the patient's disease (a 3^rd^-year nursing student)
	CF4. Checking the extent of IC practice based on the routes of transmission	Disposable gown is a large possibility of contact with the body when treating feces, and I thought that a gown could be easily removed and disposed on the spot; so I chose the gown; and I chose a regular mask as the pathogen is not transmitted through the air, so it would be okay to use a regular mask that would only cover the nose and mouth (a 4^th^-year nursing student)
	CF5. Considering the interactive dynamics of the pathogen and its host	It is unavoidable for this patient to be in a negative pressure room due to chickenpox; however, the patient had a bone marrow transplant 6 months ago and currently has a fever condition; the patient's immunity is considerably weakened. Hence, the following two points should be considered together. I should be careful not only to make sure that this patient does not infect other patients, but also to make sure that this patient is not infected by other patients (a 4^th^-year nursing student)
	CF6. Categorizing patients to prioritize IC practice for multiple patients care	Infected patients have to perform more in a limited time compared to other patients. Hence, I have to think about how to do it efficiently. This patient is important, but I think of other patients as well. When nursing an elderly patient with low immunity, do not touch them immediately without hand hygiene after contact with the infected patient. Of course, this patient should not be prioritized over other patients, and other patients should not be prioritized, but I think we should think about how to prevent the spread of the infection among many patients (a 3^rd^-year nursing student)
Nursing students' learning regarding IC	CL1. Mechanical memorization of IC principles	In this case, wearing protective equipment and single room isolation were learned in the fundamentals of nursing class. In symptomatic treatment, I learned about resistance bacteria in microbiology (a 2^nd^-year nursing student)
	CL2. Understanding the relationship between the patients and the environment	First, I got to know the negative pressure room well while investigating the negative pressure room as a group task in the fundamentals of nursing class. The group assignment had the theme of nursing for MERS patients. I learned a lot about the facts, what a negative pressure room is, and what kind of protective equipment should be worn, from the manual. In the course of the investigation, I learned about the cases where medical staff could be a vector of infection when treating patients (a 2^nd^-year nursing student)
	CL3. Understanding the relationship between the pathogen and the host’s potential	Measles is highly infectious for about 4 days after the rash appears; so you need to be careful during that period. However, I thought that it was a situation where both the mother and the visitor of the patient needed to pay attention because the child is in a stage of presenting rashes (a 4^th^-year nursing student)
	CL4. Linking to IC cases in clinical practice	First of all, it seems that the knowledge I gained from the fundamentals of nursing class regarding contact isolation was the basis, and then I saw a hepatitis A patient in clinical practice. Hence, I was able to observe the patient's diet management. While studying the causes of liver cancer in the adult nursing theory class, I learned about care of hepatitis A, B, and C, and it seems that I learned more about the patient's nursing as seen in clinical practice (a 4^th^-year nursing student)
	CL5. Understanding of IC through comparisons in clinical practice	When I practiced at the MICU, my mentor was a very smart nurse, so she was able to manage infections very thoroughly. When the mentor changed, I was able to compare a nurse who was thorough in IC with a nurse who was not. Thus, I realized that a patient could be infected by the careless behavior of the nurse in a clinical setting (a 4^th^-year nursing student)
	CL6. Breaking the chain of infections	In the case of this patient, the test for measles was negative; however, I think there may be two situations in which there may be other germs or just a fever. If the situation involves contamination by a germ other than measles, prescription for fever is required. It seems that you can think about discharge from the hospital depending on whether the symptoms improve after administering the medicine that has been used (a 3^rd^-year nursing student)
	CL7. Understanding the overall hierarchy of IC within nursing units	I have to do all of the protective isolation, and I think I will prioritize isolating the patient. When patient protection is professional and ethical, it also protects other patients, and as a protective isolation for this patient, the medical staff just cares about the infection more. I think it is more effective for this patient to get high absolute neutrophil count or catch chickenpox quickly (a 4^th^-year nursing student)

- *CF1. Structuring the spatial separation of infected patients*

This category represents the perspective of dealing with infected patients by separating them from other patients according to pathogenic characteristics in order to protect non-infected people.

- *CF2. Patterning IC protocol according to the pathogenic characteristics*

In this category, nursing students examine the transmission range of infectious agents in the clinical environment and construct a pattern that applies IC principles in nursing and treatment.

- *CF3. Identifying the possibility of infection during the incubation period*

Nursing students identify people who have had contact with the patient during the incubation period and those who are likely to meet the patient in the future to prevent transmission.

- *CF4. Checking the extent of IC practice based on the routes of transmission*

This category involves practicing IC based on the routes of transmission with various pathogens.

- *CF5. Considering the interactive dynamics of the pathogen and its host*

Nursing students consider how to manage infections, focusing on the immune functions of patients. In CF5, nursing students first check the conditions of a patient’s susceptibility to infection and then determine the pathogen’s invasion capability and transmission conditions.

- *CF6. Categorizing patients to prioritize IC practice for multiple patients care*

Nursing students preferentially manage infected patients in the context of caring for multiple patients in a clinical situation by considering the transmission attributes of infectious pathogens and the patients’ immune function.

### How did you learn about the IC for this situation?

We based the following seven categories of nursing students’ learning regarding IC on how they learned about IC. Key quotes of participants from each category are presented in Table 2.

- *CL1. Mechanical memorization of IC principles*

Under this category, nursing students simply memorized the theoretical content underlying IC theory and methods.

- *CL2. Understanding the relationship between the patients and the environment*

In CL2, nursing students used their experiences with similarly infected patients and related environmental factors.

- *CL3. Understanding the relationship between the pathogen and the host’s potential*

This category suggests that nursing students understand pathogens with which they are theoretically familiar in the context and the patients’ and caregivers’ potential ability to defend against them.

- *CL4. Linking to IC cases in clinical practice*

Nursing students link their newfound knowledge about IC with practical nursing tasks or with the experience of observing nurses perform such tasks when learning IC in a theoretical course.

- *CL5. Understanding of IC through comparisons in clinical practice*

This category indicates that nursing students have learned about IC by comparing and reflecting on infection-related practices among practitioners.

- *CL6. Breaking the chain of infections*

Nursing students explain IC by applying the cases of patients observed in a clinical setting to break the chain of infections and by simulating situations that could actually occur.

- *CL7. Understanding the overall hierarchy of IC within nursing units.*

Nursing students can organize nursing priorities by implementing a management hierarchy of infected patients within a nursing unit according to the infectious pathogens.

### What is the structural framework of identified categories of how nursing students learn IC?

A fundamental belief of phenomenography is that the experience of any phenomenon can be meaningfully categorized, and the number of categories is limited and determinable [22]. In phenomenography, results are described in terms of categories and relationships between categories and are represented by outcome spaces. The outcome spaces can be divided into referential and structural aspects. The referential aspect describes the varying meanings of the conception of a phenomenon, whereas the structural aspect describes how each category is related hierarchically [21].

**Fig. 1:**
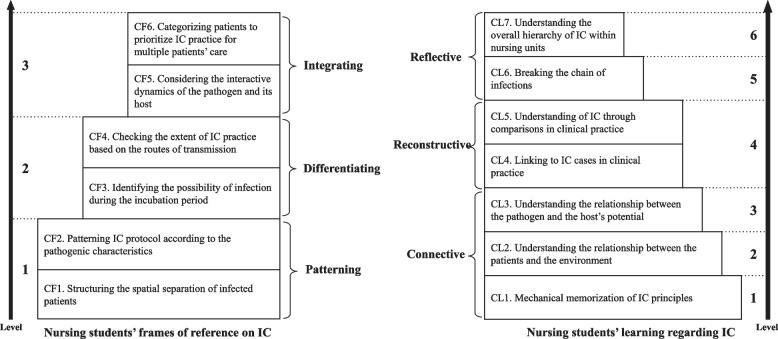
The structure of description categories related to infection control (IC) by nursing students

We divided the referential aspects of the categories of nursing students’ frames of IC care into patterning, differentiation, and integration. Nursing students’ learning regarding IC involved connecting, reorganizing, and reflecting. These aspects formed the structural framework of nursing students’ IC education during undergraduate nursing programs. A phenomenographic study yields the possibility of viewing the outcome as a collective, comprehensive, and accurate picture of the possible ways of experiencing a phenomenon [29]. Through this study, the outcome of how nursing students conceptualize and learn IC care is presented in Fig. 1.

## Discussion

IC is an integral part of the day-to-day work of health professionals to protect patients and healthcare professionals [30, 31]. In addition, hospitals are building a culture of patient safety, and as the most essential factor for patient safety, IC is important in constructing a safe hospital environment [32, 33]. Students’ IC practices were positively influenced primarily by their university education and the good practices of their clinical mentors [34]. This study aims to explore how nursing students conceptualize and learn IC care from the learner’s perspective using phenomenography. To inquire about the nature of learning, it is necessary to understand and analyze learning from the learner’s point of view.

This study found that, with regard to the frames of reference for IC care adopted by nursing students, the referential aspects were patterning, differentiation, and integration. In general, nursing students follow a principled management pattern in IC that distinguishes between infected and non-infected patients, as they acquire the concept of identifying those who may be infected to prevent the spread of infection. For example, one participant shared: “I think IC care is a measure that can protect general patients and other people who are not infected first.” Only some of the 3^rd^- and 4^th^-year nursing students who experience clinical practice tend to identify infected patients by considering the nursing unit’s circumstances and the overall patients in the unit that may lead to the spread of infections. To form a higher conceptual frame of reference of IC care, nursing educators need to apply a constructivist theoretical approach that emphasizes the learner’s activities in the knowledge-building process [35] to IC nursing education. In other words, nursing educators are required to plan a curriculum that can gradually improve the frames of reference of IC care by using various teaching methods such as problem-based learning, so that students can be exposed to different perspectives in the classroom. In addition, to integrate the frame of reference of IC care for nursing students, it would be helpful to not only emphasize the practice of IC care according to the transmission condition of infectious agents but to also provide a perspective on the hierarchy of IC care based on the unit environment and multi-patient contexts.

The study’s findings about nursing students’ learning regarding IC show that the referential aspects were connection, reconstruction, and reflection. KA Fletcher, VL Hicks, RH Johnson, DM Laverentz, CJ Phillips, LNB Pierce, DL Wilhoite, and JE Gay [36] identified five major themes as the defining attributes of conceptual learning: recognizing patterns in information, forming linkages with a concept, acquiring a deeper understanding of a concept, discovering personal relevance and constructing value to self, and applying concepts to other situations. Nursing students’ learning, regardless of their university year, was centered on making connections using the theoretical IC knowledge they had memorized mechanically during the fundamentals of nursing courses. Only a few 3^rd^- and 4^th^-year students who had experience with clinical practice were learning to compare and analyze IC knowledge and reconstruct it in clinical situations and to apply IC knowledge in consideration of the condition of nursing practice. Similar to a previous study [3], these findings imply that nursing students learn about IC care differently before and after clinical practice and that the depth and focus shift as nursing students progress from a lower to a higher academic year. However, not all 3^rd^- and 4^th^-year students who experienced clinical practice had the category of reconstruction and reflection in IC learning. Therefore, nursing educators should avoid assuming that nursing students will properly understand IC care and that the reference aspects of learning will be developed through the academic year. Theoretical education based on the abundant clinical cases is required to promote the integrated and reflective IC learning of nursing students. Additionally, educational opportunities should be sufficiently provided in a manner that connects clinical cases with IC knowledge and reconstructs and reflects IC knowledge from a critical perspective.

In this study, nursing students’ IC care was categorized into six frames of reference and seven categories of learning. Even in the same academic year, differences were drawn in conceptualizing and learning IC care. Cox et al. (2014) [14] described that it is difficult for clinical mentors, as they guide students in a variety of clinical settings, to estimate what level of IC education their students are receiving. Moreover, according to a study by EJ Carter, D Mancino, AJ Hessels, AM Kelly, and EL Larson [6], 89% of respondents reported that there is an agreement between what they are taught in school and what they observe in clinical rotations, yet 51% of respondents noted that they had witnessed poor practices related to IC in their clinical placement. In general, nursing colleges in Korea integrate IC training throughout their student curriculum and do not offer dedicated IC courses. Consequently, nursing educators who are well versed in individual nursing courses may not be aware that they are also responsible for teaching IC [3]. In the nursing classroom, theoretical IC principles are emphasized, but in clinical practice and training, IC care is applied by considering the environment and task hierarchy. A lack of consistency in improving students’ learning and competency through instructional method and practice may be the missing link [37]. Therefore, a consistent and dedicated IC education course that includes various clinical situations can lead to standardizing nursing students' IC conceptualizations and a more progressive IC education.

IC is an indispensable part of the routine work of health professionals to protect patients [30]. Through these findings, it can be inferred that the theoretical education on IC care in nursing students’ classrooms needs to be further strengthened as it forms the basis of nursing students’ IC reasoning in clinical settings. EJ Carter, D Mancino, AJ Hessels, AM Kelly, and EL Larson [6] suggested that better coordination is needed between classrooms and clinical settings for IC training, and that additional IC education is necessary. Considering that graduated nurses are the first line of defense to keep patients safe, it is important to note how nursing students conceptualize and learn IC in order to provide the highest level of IC care.

These findings emerge as important data to understand how nursing students conceptualize and learn IC care. Based on the results, professors and clinical mentors need to construct IC theoretical education and clinical practice considering that nursing students conceptualize IC differently and are at various levels of learning.

The data used in this study were obtained from a convenient sample of undergraduate nursing students from five universities in South Korea; therefore, participants may not be representative of undergraduate nursing students in other universities or locations. However, this study did not aim to generalize the characteristics of the phenomenon but rather to identify the nature and IC learning structure of undergraduate nursing students through phenomenographic study.

## Conclusions

To understand the nature of learning as a phenomenon, it is necessary to understand and analyze learning from the learner’s point of view. This study offers nursing educators insight into how infection control education is actually being acquired in undergraduate nursing programs from the learner’s point of view. To our knowledge, this study is the first attempt to explore the structure of nursing students’ infection control care learning from the learner’s point of view. We believe that this study presents data that nursing educators can refer to about infection control education in undergraduate courses that nursing students receive before entering the clinical setting as a nurse.

### Supplementary Information

**Additional file 1.** Interview guidelines. 

## Data Availability

The datasets generated and analyzed for the current study. Therefore, the datasets are not publicly available but are available from the corresponding author upon reasonable request.

## Abbreviations

IPC: Infection prevention and control
IC: Infection control
Dx: Diagnosis
CPE: Carbapenemase-Producing Enterobacteriaceae
MICU: Medical Intensive Care Unit
Tb: Tuberculosis
CDI: Clostridium Difficile Infection
ER: Emergency Room
PCR: Polymerase Chain Reaction
