# Supplementary material for: How nursing students learn infection control education through undergraduate nursing programs: a phenomenographic research study
Source: BMC Nurs. 2023 Aug 31;22:297. doi: 10.1186/s12912-023-01465-9 (PMC10470169; doi:10.1186/s12912-023-01465-9)
Supplement: Supplementary file 1 — Additional file 1. Interview guidelines. [file 12912_2023_1465_MOESM1_ESM.docx]

Additional File 1. Interview Guidelines

| ***Step 1.*** | - Describe the research topic, the aims, and method of interview - Obtain permission to record - Prepare nine vignettes on a separate page and provide them to students sequentially in numerical order | |
| --- | --- | --- |
| ***Step 2.*** | **Main interview questions (Q)** | **Rationale for the question** |
|  | - **Start interview by presenting the first vignette paper to the student.**   **I. The first vignette**   - Wait until the student reads and understands the vignette completely. - Next, ask students to define the situation in their own words. *“Please summarize the first vignette…”* | - Provide time to read the presented vignette and organize thoughts. - Ensure that the presented situation is properly understood and defined. |
|  | - ***Q1****.* If you were a nurse, could you tell me what kinds of nursing practices you would engage in based on the scenarios related to the three types of isolation? *“What kind of isolation guidelines should be followed for managing the patient’s room and caregivers’ education?” “What kinds of nursing practices should be performed on this person?”* | - Classify the types of isolation based on the disease and patient condition presented in the vignette, and ask what kinds of nursing practices are needed. - Guide students to framing themselves within the presented clinical situation and conceptualize how to perform IC care. |
|  | - ***Q2****.* What kinds of nursing practices would you undertake to implement infection control? *“As a nurse in charge of a patient with active tuberculosis, what infection control* *should be taken for room management and caregivers’ education?”* | - Provide students specific questions to perform nursing practices for IC according to the type of isolation based on their own knowledge of IC care. - Lead students to express their perception on IC care regarding Q2. |
|  | - ***Q3****.* What knowledge or experience is the foundation of current infection control nursing practices? *“You said that* *the patient should be in a negative pressure room and that the caregivers should be informed that it is difficult to visit in person. What knowledge or experience has this answer been based on?”* | - **The key questions of this study** - Encourage students to express how they came to know the answer to Q2 and how they came to think of IC care in the answer to Q2. |
|  | - **Ask additional probing questions as needed**   *“The three types of isolation are…”* | - If necessary, provide additional information so that the students can clearly understand the main interview questions. |
|  | **Ⅱ. Continue the interview in the same manner with other vignettes from the second to ninth consecutively.** | - Guide students to conceptualize IC care independently through the nursing education curriculum by interpreting and analyzing the IC situation presented in the nine vignettes. |
| ***Step 3.*** | - **Rephrase answers and ask additional questions**   *“So does that mean…?” “Can you give me an example of…”* | - Provide sufficient opportunities to the students to reflect and consider the knowledge and experiences of IC care in the nine vignettes. |
| ***Step 4.*** | - **Summarize and close the interview**   *“Is there anything else you would like to say about this interview?”* | - Ensure that the student responds comprehensively on the topic and that there are no additional comments. |
